# The influence of platelet-rich plasma (PRP) on colonic anastomosis healing impaired by intraperitoneal 5-flourouracil application. An experimental study

**DOI:** 10.1590/s0102-865020200050000004

**Published:** 2020-07-06

**Authors:** Mustafa Gorur, Alper Sozutek, Oktay Irkorucu, Burak Karakaya

**Affiliations:** I MD, Department of General Surgery, University of Health Sciences Adana City Training and Research Hospital, Adana, Turkey. Analysis of data, technical procedures, manuscript preparation.; II PhD, Associate Professor, Division of Gastroenterological Surgery, Department of General Surgery, University of Health Sciences Adana City Training and Research Hospital, Adana, Turkey. Technical procedures, design of the study, analysis of data, manuscript preparation.; III PhD, Professor, Department of General Surgery, University of Health Sciences Adana City Training and Research Hospital, Adana, Turkey. Analysis of data, critical revision.; IV MD, Department of General Surgery, University of Health Sciences Adana City Training and Research Hospital, Adana, Turkey. Acquisition of data.

**Keywords:** Anastomosis, Surgical, Platelet-Rich Plasma, Drug Therapy, Flourouracil, Rats

## Abstract

**Purpose:**

5-flourourasil (5-FU) is commonly used for early intraperitoneal chemotherapy in colorectal or appendiceal cancer patients with peritoneal carcinomatosis. Due to its effect, anastomosis healing can be impaired and leads to anastomotic leakage. In this study, we aimed to investigate the potential healing effect of platelet-rich plasma (PRP) on colonic anastomosis impaired by intraperitoneal 5-flourouracil application.

**Methods:**

After ten rats were sacrificed for preparing PRP, forty Wistar-albino rats were subjected to colonic anastomosis, and randomly allocated into four groups including 10 rats each. According to receiving PRP and/or 5-FU application, the groups were formed as control (C), 5-FU without PRP (CT), anastomosis with PRP (C-PRP), and 5-FU with PRP (CT-PRP). CT and CT-PRP groups also received 5-FU intraperitoneally on postoperative day 1 (POD 1). All animals were euthanized on pod 7. The body weight change, anastomotic bursting pressure (ABP), tissue hydroxiprolin (TH) and histopathological examination of each group were analyzed.

**Results:**

5-FU application significantly reduced ABP levels when compared with group C, C-PRP and CT-PRP (for each comparison, p<0,01). PRP application in CT-PRP group raised the measure of ABP up to the levels of C group. Although tissue hydroxyproline levels (THL) levels of CT-PRP group were found higher than CT group, it was not significant (p=0.112). Microscopically, comparing with CT group, PRP application significantly promoted the healing of colonic anastomosis subjected to 5-FU application by improving tissue edema, necrosis, submucosal bridging and collagen formation (p<0.05). Tissue healing in CT-PRP group was observed as good as the control groups. (C, C-PRP, p=0.181, p=0.134; respectively).

**Conclusion:**

PRP administration on colonic anastomosis significantly promotes the healing process of anastomosis in rats receiving 5-FU. This result encourages further clinical use of PRP to reduce the frequency of AL in patients receiving EPIC.

## Introduction

Cytoreductive surgery (CRS) with intraperitoneal chemotherapy (IPC) is considered as an aggressive surgical technique; however, it yields potential oncological results for colorectal or appendiceal cancer patients with peritoneal dissemination^1-3^. Although many postoperative complications may occur after such an aggressive surgery, anastomotic leak (AL) is the most dreaded one which leads to significant morbidity and mortality^4,5^. Being sure of that the most of the surgical team is precisely performed the CRS+IPC operation, unwilling temporary/permanent stoma is created because of their justified postoperative concerns. AL rates have been reported higher in CRS+IPC compared to conventional colorectal surgery ranging from 10% to 25% in various studies^4-8^. Intraperitoneal administration of cytotoxic drugs negatively affects the healing of colonic anastomosis likely due to impair the postoperative collagen synthesis^9-11^.

Despite many advances in surgical equipment and techniques, AL after colorectal surgery still remains a major problem for the surgeons yet to be solved even under favorable conditions. Because AL is related to multifactorial conditions including patient characteristic, surgical technique, tumor biology, administered medicine etc., it seems the debates and also the efforts will continue for a long time to prevent this complication^12,13^. Among these factors leading to AL what surgeons can act directly is to strengthen and also facilitate to improve the healing of the anastomotic line.

Platelet rich plasma (PRP) is known as an autologous platelet concentrate suspended in plasma which contains multiple growth factors. When platelets become activated, these growth factors are released and accelerate wound healing process 15. In fact, promising results about the colonic anastomotic healing effect of PRP application on colonic anastomosis has been reported in previous studies including us^15-16^. Our results encouraged us to design a novel experimental model. Recently, its anastomotic healing effect has been evaluated by two similar studies regarding intraperitoneal administration of oxaliplatin and cisplatin with HIPEC^17,18^. However, 5-FU is commonly used for early EPIC^1^. To our knowledge, there is no study evaluating the effect of PRP on the healing of colonic anastomosis impaired by intraperitoneal 5-FU administration to date.

This study was designed as an experimental model because of the difficulty of designing a controlled, randomized, prospective clinical study. The purpose of this study was to investigate whether PRP has a positive effect on the healing process of colonic anastomosis impaired by early intraperitoneal 5-FU application.

## Methods

This study was conducted at the Experimental Research Center of Mersin University, Mersin, Turkey after obtaining the ethical committee approval of Mersin University Medical Faculty (Approval number: 2016/10). Fifty Wistar-albino male rats, weighing 180 to 225 g were used in the present study. The animals were maintained at 21 ^0^C, humidity at 40%-60% with a 12 h light/dark cycle and were allowed free access to water and standard chow during the study. All animals were observed closely and weighed on days 7 after surgery. This research was carried out in accordance with the Guide for the Care and Use of Laboratory Animals (NIH, 1985).

### *PRP preparation/activation and medication*

PRP was prepared followed by two-stepped centrifuge of whole blood taken from healthy rats. Nine milliliters of whole blood were drawn from 10 healthy rats through cardiac puncture into ten 10 ml PRP tubes (Cence Medical, Istanbul, Turkey) containing 0.5 mL buffered sodium citrate with a ratio 9:1. Centrifuge levels were set according to the manufacturer’s instruction (Cence Mecical). The whole blood was centrifuged 400 x g and 20^0^C for 10 min. Blood was separated into three layers as; red blood cells at the bottom, buffy coat layer in between, and acellular plasma in the supernatant. Subsequently, the supernatant was transferred with a sterile pipette to another 10 ml centrifuge tube and re-centrifuged at 800 x g and 20^0^C for 10 min. About 1ml of PRP was collected from the bottom of the tube. To activate PRP and obtain a viscous coagulum gel that can be applied to anastomosis, 1 mL of PRP was mixed with the activator including 50 µL of 10% calcium chloride (B. Braun Medical SA) for neutralizing the anticoagulation effect of the citrate and 1mL of thrombin (Diamed, Morat, Switzerland (PPT-Reagent) to initiate the clotting process.

5-FU (500 mg/10 ml I.V, Kocak Farma, Istanbul, Turkey) was diluted with 3 ml saline and was prepared as a solution of 10 mg/ml to resemble the human dose recommended for application.

### *Study design/surgical procedure*

Whole blood was drawn from 10 healthy rats through cardiac puncture to prepare PRP. The rest of the rats were randomly allocated into four groups of 10 rats each, as follows:

Group 1: Control group (C): subjected to colonic anastomosis without PRP administration.

Group 2: Control group after 5-FU application (CT): Intraperitoneal 5-FU administration followed by colonic anastomosis without covering with PRP.

Group 3: Control PRP group (C-PRP): subjected to colonic anastomosis covered by PRP.

Group 4: 5-FU+PRP group (CT-PRP): Intraperitoneal 5-FU administration followed by colonic anastomosis covered by PRP.

After an overnight fast, the rats were anesthetized by intramuscular injection of ketamine 50 mg/kg (Ketalar; Parke Davis, Eczacibasi, Istanbul,Turkey) and xylazine 10 mg/kg (Rompun; Bayer AG,Leverkusen, Germany). Animals were allowed to breathe spontaneously during the surgery. A heating lamp was used to preserve the body temperature at 37^0^C. All operations were performed by the same surgeon to ensure technical uniformity. The abdominal skin of the rat was shaved, and under aseptic condition a 4 cm midline incision was made. In all groups, the left colon was resected approximately 2.5 to 3.5 cm above the peritoneal reflection and an end to end hand sewn anastomosis was performed using one layer of interrupted 4/ 0 silk sutures. In PRP groups, activated 0.5 mL PRP gel was applied to the anastomosis as a film layer of 2mm width and 1mm thickness. In group 2 and 4, 5-FU was administered intraperitoneal by two catheters after creating the anastomosis. The inflow catheter was replaced to the subdiaphragmatic space and the outflow catheter was placed in the pelvis. After closing the abdomen, 20mL/kg 5-FU in 150 mL saline was administered and dwelled for 60 minutes. Subsequently, it was drained from the outflow catheter. The muscle-fascial layer was closed with a single layer continuous absorbable 3/0 suture (Vicryl, Johnson & Johnson) and the skin was closed with interrupted 3/0 silk suture.

Postoperative course was assessed every 6 h within the first 48 h and every 8 h for 5 days. Postoperative analgesics (Diclofenac sodium 2 mg/kg, I.M) were used in postoperative day 1 (POD) since to control symptoms associated with pain including discomfort, agitation or itching of wound side. Group 2 and 4 was also received a dose of 20 mL/kg 5-FU 1 cm away from the incision line intraperitoneally, on POD 1.

**Figure 1:**
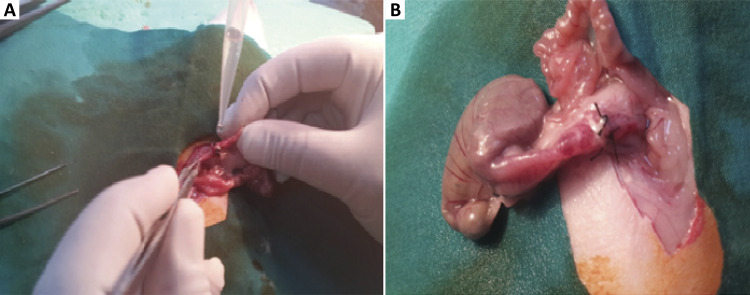
Surgical procedure. A) PRP application on colonic anastomosis. B) The view of colonic anastomosis in CT-PRP group on postoperative day 7.

On postoperative day 7 (POD 7), all animals were sacrificed by intraperitoneal overdose 2ml pentobarbital sodium (200 mg/ml, KU Life, Copenhagen). The anastomosis was carefully found in order to avoid interruption of the mesenteric blood supply or adhesions to determine the exact anastomotic bursting pressure *in vivo* (Fig. 1). The anastomosis was resected with a margin of at least 3 cm on each side with regard to histopathologic examination and tissue hydroxyproline level followed by measuring the anastomotic bursting pressure.

### *Anastomotic bursting pressure (ABP) measurement*

The abdomen was opened under anesthesia and ABP was measured *in vivo* to evaluate the integrity and strength of the anastomosis. A 14-gauage silicon catheter was inserted from both sides of the anastomosis with a distance of 3 cm followed by cleaning feces with isotonic saline. Both sides were occluded with 3/0 silk suture. Isotonic saline was administered at 4 ml/min rate with an infusion pump while the pressure within the lumen was monitored via the transducer of a pressure monitoring system connected to the other catheter. ABP was defined as the pressure at which saline leak was observed and corresponded with the maximum pressure attained just before rupture of the anastomosis.

### *Histopathological examination*

A 1 cm-long sample including colonic anastomosis was immediately fixed in 4% formaldehyde. The samples were dehydrated, embedded in paraffin and cut in 4-µm thick slices. Histopathologic sections were stained with hematoxylin-eosin (HE) for inflammation parameters and picrosirius red for collagen. A conventional binocular Leica DM 2000 light microscope (Leica Microsystems, Wetzlar, Germany) was used for analysis. The samples were assessed in a blinded manner by two pathologists to avoid bias.

Inflammation parameters including necrosis, polymorphonuclear cells, lymphocytes, macrophages, edema, state of epithelial layer, and bridging of submucosal-muscular layer were scored according to Verhofstadt scale^19^. Collagen formation was assessed as decreased deposition (0), normal (1) or increased deposition (2).

### *Hydroxyproline level measurement*

After measuring ABP, 1 cm of the anastomosis including 0.5 cm proximal and distal from the anastomosis were excised and blunt dissection was performed to clear the anastomotic line from adherent tissues. The concentration of hydroxyproline was measured using a modified procedure based on alkaline hydrolysis of the tissue and subsequent determination of the free hydroxyproline in hydrolysate^13^. Chloramine- T was used to oxidize the free hydroxyproline for production of a pyrrole. The addition of Ehrlich reagent resulted in the formation of a chromophore that can be measured at 550 nm. Results were expressed as micrograms of hydroxyproline per gram of dry tissue (µg/mg, dry tissue).

### *Statistical analysis*

Statistical Med Calc 9.3.9.0 software was used for sample size calculation. The primary outcome variable was anastomotic bursting pressure with a different rate of 10 % between the groups. Using a power, 80% α-error and 5% β-error, a sample size of 8 rats were calculated for each group to show a statistically significant difference. Considering the possible deaths during the study, 10 rats were allocated for each group. The data obtained were summarized in a computerized spreadsheet and statistical analyses were performed by using SPSS 18.0 for Windows. Numerical data were presented as mean ± standard deviation (SD). The mean values of ABP, the levels of tissue hydroxyproline and histological scores were analyzed with using one-way analysis of variance (ANOWA) and Tukey’s HSD post-hoc test were used to assess the differences between the groups. Differences were considered statistically significant at *P* < 0.05.

## Results

### *General observations*

All animals survived surgery. No local or systemic complications related to PRP or 5-FU administration were observed. The initial body weight was almost similar in four groups. Although the mean body weights were reduced in all groups during the study period, there was no statistical difference among the groups in terms of body weight changes on POD 7.

#### Anastomotic bursting pressure

**Figure 2:**
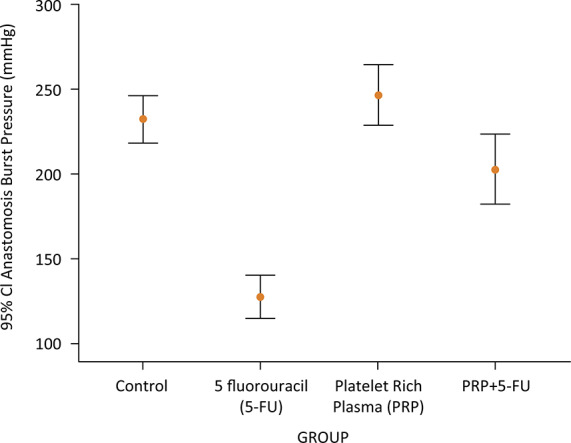
The median levels of anastomotic bursting pressure (ABP) according to the groups.

The anastomotic bursting pressure was measured at a mean value of 232.6 ± 19.5, 127.5 ± 17.7, 246.7 ± 25.1 and 202.9 ± 28.8 mm-Hg, in group C, CT, C-PRP and CT-PRP; respectively. Considering the results, 5-FU application significantly reduced ABP levels when compared with group C and C-PRP (127.5 ± 17.7 *vs*. 246.7 ± 25.1, 246.7 ± 25.1, p <0.05; respectively). Although it was not significant, PRP application on healthy intestinal anastomosis (C-PRP) increased the ABP when compared with group C (232.6 ± 19.5 *vs*. 246.7 ± 25.1, p>0.05). Furthermore, it is notable that PRP application in CT group (CT-PRP) raised the measure of ABP up to the levels of C group (232.6 ± 19.5 *vs*. 202.9 ± 28.8, p=0.128). It was also significantly higher when compared with CT group (202.9 ± 28.8 *vs*. 127.5 ± 17.7, p<0.05). These results revealed that PRP application significantly improved the strength and the integrity of colonic anastomosis in case of CT-induced reduction of ABP (Fig. 2).

#### Tissue hydroxyproline levels

**Figure 3:**
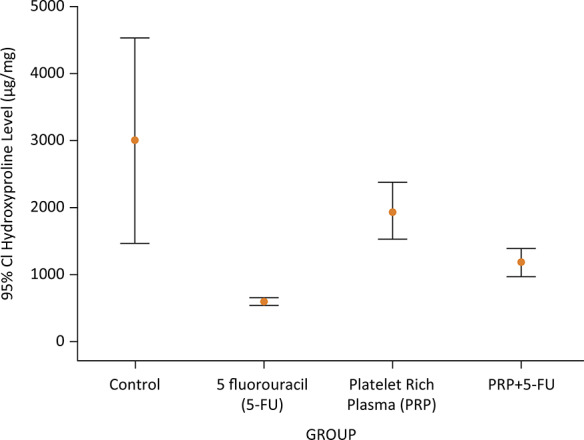
The median levels of tissue hydroxyproline (THL) according to the groups.

Tissue hydroxyproline levels (THL) were measured at a mean value of 2994.6 ± 2132.4, 591 ± 84.4, 1939.5 ± 586, and 1171 ± 301.7 µg/gr in group C, CT, C-PRP and CT-PRP; respectively. THL was significantly lower in CT-group when compared with C and CT-PRP groups (p <0.05). Although THL levels of CT-PRP group were found higher than CT group, it was not significant (p=0.112). Surprisingly, THL of C-PRP group was found lower than C-group; however, it was not significant (p=0.212). Despite confusing results, these data revealed the positive effect of PRP on THL of colonic anastomosis (Fig. 3).

The body weight change, anastomotic bursting pressure and hydroxyproline levels of the groups were summarized in Table 1.

**Table 1:** Body weight change, anastomotic bursting pressure and hydroxiprolin median levels of the groups.

Parameters	Group A Control (n=10)	Group B 5-FU (n=10)	Group C Control PRP (n=10)	Group D 5-FU + PRP (n=10)	p value (Groups comparison)
Initial body weight (g)	196 ± 12.6	194 ±13.9	193,5±11.9	196±10.7	NS
Body weight on POD7	195±8.2	181±9.8	190±7.5	188±5.2	
Anastomotic bursting pressure (mm-Hg)	234± 19.5	127.5±17.7	251±25.1	203.5± 28.8	**p<0.001 B-A,C,D C-D**
Hydroxiprolin levels (µg/mg tissue)	2423±2132.4	609±84.3	1767±586	1153±301.7	**p<0.001 B-A,C A-D**

#### Histopathological examination

**Figure 4:**
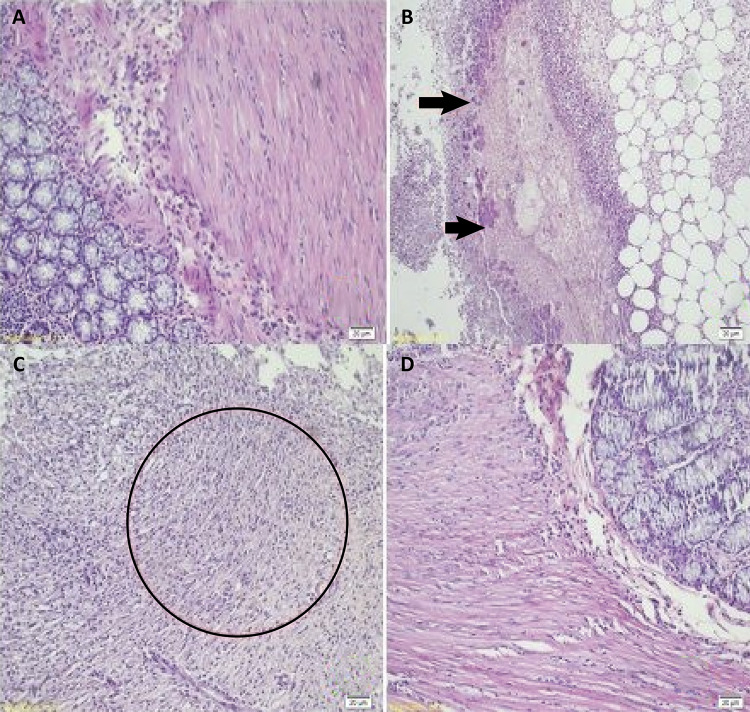
Histopathological examination of the specimen. A) Normal anastomotic healing. B) Tissue necrosis in group B. C) Impaired submucosal bridging in group B. D) Anastomotic healing of group D as in control group.

Verhofstad scale is a good scoring system for grading histological changes in the tissue that allows us to evaluate the healing on cellular base and tissue remodeling. There were statistically significant differences among the groups, particularly in terms of mucosal healing (Table 2). Although, no significantly data were recorded in terms of inflammatory parameters, significant differences were observed microscopically in CT group regarding tissue necrosis, edema and submucosal bridging. PRP application promoted the healing of colonic anastomosis subjected to 5-FU application by improving tissue edema, necrosis and submucosal bridging. Tissue healing in CT-PRP group was observed as good as the other control groups. (C, C-PRP, p=0.181, p=0.134; respectively). There was no statistically significant difference in terms of collagen formation and also other inflammatory parameters between these groups. However, although increased collagen formation was noted in CT-PRP group, no statistically significant difference was detected between CT and CT-PRP group (p=0.112) (Fig. 4).

**Table 2:** Histologic scores of the groups.

Parameters	Group A Control (n=10)	Group B 5-FU (n=10)	Group C Control PRP (n=10)	Group D 5-FU+ PRP (n=10)	p value
Necrosis	0.3 ±0.67	1±1.05	0	0.1±0.3	**0.006 B-A,C,D**
PMNs	1.3 ± 0.94	1.5±0.53	0.7±0.67	1.2±0.42	**NS**
Lymphocytes	1.0±0.47	1.4±0.32	1.0±0.67	1.5±0.58	**NS**
Macrophages	1.0+0.00	1.1±0.83	1.8±0.31	1.6±0.53	**NS**
Edema	1.1±0.31	2±0.73	1±0.47	1.7±0.48	**0.007 B-A,C,D**
Mucosal epithelium	0.3 ± 0.57	1.1±0.42	0.3±057	0.6±0.57	**0.03 B-A,C,D**
Bridging	1.2±0.78	1.5±0.52	0.9±0.87	1.1±0.31	**0.03 B-A,C,D**

## Discussion

In the present study, the anastomotic healing effect of PRP was assessed depending on the results of three valid determinant factors: anastomotic bursting pressure (ABP) for anastomotic strength and integrity, tissue hydroxiproline levels (THL) for biochemical evaluation and histopathological examination for evaluating tissue regeneration. Our data revealed that 5-FU administration significantly impaired the healing process of colonic anastomosis. The levels of ABP were significantly lower when compared with the control groups. On histopathological examination, significant necrosis and edema on anastomotic line was observed in rats that solely received IPC. It is well known that collagen formation is a main indicator of the healing process of anastomosis. THL, which is a good marker for determining the collagen concentration of the tissue, was also significantly lower when compared with the other groups. This data revealed that intraperitoneal 5-FU administration impeded the anastomotic healing in cellular base, and it was compatible with the previous studies. However, PRP administration on colonic anastomosis promoted the wound healing process, a significant increase in the levels of ABP and TH in CT-PRP group was recorded when compared with the CT group. It is notable that the mean measurement levels of ABP raised up to the levels of C group. It was likely due to the fact that PRP stimulated the collagen production of the healing tissue site, thus it contributes better tissue regeneration which provides the integrity of anastomosis in CT-PRP group.

Since Sugarbaker^21^ introduced the CRS plus HIPEC, in the early 1990s, this revolutionary approach has gained wide popularity among the surgeons to cure patients with colorectal and appendiceal malignancy with peritoneal spread. EPIC is another way of intraperitoneal chemotherapy, particularly performed in patients with incidentally detected wide dissemination of colorectal or appendix cancer which is suitable for complete cytoreduction and lack of facilities for HIPEC, as well. 5-FU is the most commonly used agent for this purpose^1^. Because 5-FU does not act synergistically with hyperthermia, we did not use hyperthermia in the present study. 5-fluorouracil (5-FU) is an antimetabolite type of chemotherapy which forms the cornerstone of several different chemotherapy regimens. 5-FU is cell cycle specific and appears very similar to normal substances within the cell, thus it interferes with cellular metabolism to prevent cell division. Decreased leukocyte accumulation, leading to reduced production of local cytokines and peptide growth factors, may cause a delayed wound healing which results in anastomotic failure^9,10,20^.

Colonic anastomotic healing involves the complex interaction of multiple peptide growth factors (PGFs) thus collagen turnover is provided through the phases of inflammation, fibrosis, and maturation. 5-FU inhibits the expression of these growth factors such as TGF- β1, IGF-1 etc., which are essential factors for collagen formation. The wound healing and tissue regeneration effect of certain growth factors such as VEGF, PDGF, TGFβ-1, 2, EGF and IGF has been discussed in several studies ^14-16^. PRP contains the concentrations of these growth factors that constitute the theoretical basis of the use of PRP in wound healing and tissue regeneration. PRP is a physiological clotting material, which provides tight attachments to the anastomotic line that enables tight scaffolds between the cells and also sustains delivery of growth factors. In addition, thrombin content in PRP plays an important role in keeping the viability of platelets, thus providing continuous secretion of viable growth factors described above. Therefore, we preferred to test the influence of PRP on the anastomotic healing process impaired by intraperitoneal, 5-FU administration. Interestingly, there is no published data evaluating the influence of PRP under such an adverse condition to date. According to our extensive literature search, this study is the first. According to our results, we concluded that PRP administration to colonic anastomosis significantly promotes the healing process by accelerating tissue regeneration and remodeling, even in anastomosis subjected to intraperitoneal 5-FU administration.

Despite the survival advantages of CRS combined with HIPEC or EPIC, these treatment modalities have higher morbidity rates which are mostly related to AL. Although the reasons of AL are multifactorial, it is clear that anastomosis is more prone to dehiscence when subjected to chemotherapy agents. Due to this fact, the majority of surgeon avoids creating intestinal anastomosis and creates stomas to prevent fecal peritonitis and its fatal results^6,7^. However, stoma-related complications including severe dehydration, electrolyte imbalance retraction, necrosis and reduced quality of life are not rare. Additionally, stoma closure surgery is also associated with a higher rate of morbidity and mortality^7^. According to our results, PRP provided us to construct safer colonic anastomosis by promoting the healing process of the colonic anastomosis subjected to intraperitoneal 5-FU administration. Moreover, it can be easily prepared and applied in the operating room in about one hour^16,17^. Because of its autologous nature, PRP is more biocompatible and is cheaper when compared with other reinforcement materials^20,22^.

There were indeed several limitations in our study. Although we tried to accommodate the number of rats by using power analysis, the small sample size was the first limitation of our study. Second limitation was that the effect of PRP on colonic anastomosis was evaluated without several factors which are associated with AL. It is notable that tissue healing may differ in the presence of other pathophysiological factors. Additionally, 5-FU is used during five days for EPIC. However, we repeated the perfusion only twice due to the toxicity of the drug and also technical difficulties. Third limitation was that PRP is described as an autologous concentrate of platelets; whereas we used homologous PRP which may lead to overt immune response. Unfortunately, in small animal models such as rats, approximately a total amount of 8-10 mL whole blood can be obtained from each rat, thus we initially sacrificed 10 healthy rats. The fourth limitation was that we evaluated the overall biological effect of PRP on anastomotic healing. Unfortunately, we could not be able to identify the specific growth factors in PRP that actually promotes the anastomotic healing because of the lack of our facilities. Although our study failed to reflect the exact clinical experience because of these limitations, we assumed that solely the healing effect of PRP on colonic anastomosis impaired by 5-FU administration was well demonstrated with this study.

## Conclusions

Our results demonstrated that intraperitoneal 5-FU administration has a significant adverse effect in the healing process of the colonic anastomosis. However, PRP administration on colonic anastomosis significantly promotes the healing process, thus increasing the integrity of anastomosis in rats receiving intraperitoneal 5-FU. Although our study was experimental, these results encourage further clinical application of PRP on colonic anastomosis to reduce the frequency of AL in patients receiving EPIC with 5-FU. However, this suggestion needs to be supported by controlled, randomized, prospective clinical studies.

## Footnotes

1 Research performed at Experimental Research Center of Mersin University, Turkey.
